# Differences in Nectar Traits between Ornithophilous and Entomophilous Plants on Mount Cameroon

**DOI:** 10.3390/plants10061161

**Published:** 2021-06-08

**Authors:** Štěpán Janeček, Kryštof Chmel, Francis Luma Ewome, Karolína Hrubá, Yannick Klomberg, Ishmeal N. Kobe, Raissa Dywou Kouede, Jan E. J. Mertens, Marcus Mokake Njie, Robert Tropek

**Affiliations:** 1 Department of Ecology, Faculty of Science, Charles University, Viničná 7, CZ-128 44 Prague 2, Czech Republic; k.chmel@seznam.cz (K.C.); yannickklomberg@gmail.com (Y.K.); kobe.ishmeal@gmail.com (I.N.K.); jan.mertens70@gmail.com (J.E.J.M.); robert.tropek@gmail.com (R.T.); 2 Biology Centre, Czech Academy of Sciences, Branišovská 31, CZ-370 05 České Budějovice, Czech Republic; karolina.hruba93@seznam.cz; 3 Bokwango, Buea, Southwest Region, Cameroon; lumaescobar@yahoo.com; 4 Department of Zoology, Faculty of Sciences, University of South Bohemia, Branišovská 1760, CZ-370 05 České Budějovice, Czech Republic; 5 Naturalis Biodiversity Center, Darwinweg 2, 2233 CR Leiden, The Netherlands; 6 Department of Nursing, Faculty of Health Science, Herzing University, 1865 SR 436, Winter Park, Orlando, FL 32792, USA; scurai@yahoo.com; 7 Department of Zoology and Animal Physiology, Faculty of Science, University of Buea, Buea 63, Cameroon; marcusmokake9@gmail.com

**Keywords:** Afrotropics, fructose, glucose, insect, nectar, phylogenetic signal, plant–pollinator interactions, pollination syndrome, sucrose, sunbirds

## Abstract

Despite a growing number of studies, the role of pollinators as a selection agent for nectar traits remains unclear. Moreover, the lack of data from some biogeographic regions prohibits us from determining their general importance and global patterns. We analyzed nectar carbohydrate traits and determined the main pollinators of 66 plant species in the tropical forests of Mount Cameroon (tropical West Africa). The measured nectar traits included total sugar amounts and proportions of sucrose and hexoses (i.e., glucose and fructose). We report the nectar properties for plants visited by five pollinator groups (bees, butterflies, moths, hoverflies, and specialized birds). Our results indicate that, rather than specific evolution in each of the five plant groups, there was a unique nectar-trait evolution in plants pollinated by specialized birds. The ornithophilous plants had a higher proportion of sucrose and produced larger sugar amounts than the plants pollinated by insects. We also demonstrated a significant phylogenetic signal in the nectar properties in some lineages of the studied plants.

## 1. Introduction

Nectar represents one of the most important flower rewards for pollinators of zoophilous plants. During plant evolution, nectar became much more common than other plant exudates that have different primary functions than pollinator rewards [1,2,3]. Although nectar is a liquid with a complex composition [4], its main compounds are carbohydrates, assuring its high energetic value [5].

Nectar-producing organs, namely, nectaries, are usually supplied by phloem [6,7]. Nevertheless, although nectar metabolites are similar to those in phloem sap, their amounts can largely differ [8]. There are two interesting features of nectar carbohydrate composition: (1) nectar contains much higher amounts of hexoses (glucose and fructose) than phloem sap and (2) the ratio between sucrose and hexose in nectar does not reflect simple sucrose hydrolysis and differs from the 1:1 ratio. The high concentration of hexoses is caused by the hydrolysis of sucrose before its excretion by cell wall invertases [9], whereas the glucose:fructose ratio is modified in various metabolic pathways [10]. The carbohydrate composition can also be modified by the activity of enzymes secreted as nectar compounds [11] or by organisms living in nectar [12].

Despite the common characteristics of nectar carbohydrates (e.g., the dominance of sucrose, glucose, and fructose; however, see Jackson and Nicolson [13]), several empirical studies have demonstrated relatively high interspecific variability. The most discussed hypothesis behind this variability expects its evolution to have followed the requirements of different pollinator groups [14,15]. Nevertheless, selection pressures can also be mediated by other non-pollinating biotic (e.g., herbivores [16]) and abiotic (e.g., climate [17]) agents. Furthermore, nectar properties can also be correlated with other floral traits (e.g., floral shape [18]). The relative importance of pollinators for nectar adaptations may differ between individual nectar characteristics. Nectar production and sugar concentration are considered traits with high heritability [19,20]; these traits are under strong pollinator-mediated selection and are involved in the definition of pollination syndromes [21]. However, the heritability of the carbohydrate composition of nectar is more uncertain. Some previous studies found significant differences in the nectar compositions of plants in relation to particular pollinator syndromes [22,23]. Some other studies reported rather low differences or even high similarities among the pollination syndromes [15,17,24,25,26], whilst others did not find any significant relationships between the pollination syndromes and nectar composition [27,28]. Conclusions on the typical composition of nectar carbohydrates for the particular pollination syndromes are not consistent throughout the literature. However, consistent results were detected in hummingbird-pollinated plants, which contain a relatively high proportion of sucrose [26]. For specialized sunbirds, such a pattern was nevertheless demonstrated almost exclusively out of Tropical Africa (e.g., [29] and citations therein; however, see [30]), the area with the highest number of sunbird species [31]. In parallel, nectars of the chiropterophilous plants are characterized by a high proportion of hexoses [15,24,26]. On the other hand, nectars produced by plants of other pollination syndromes, for example, myophilous or melittophilous plants, can be highly species-specific [26].

Nevertheless, the interspecific carbohydrate nectar composition variability was often found to be lower among phylogenetically related species. Such a phylogenetic signal has been demonstrated in many plant lineages. Sucrose-dominated nectars were found in the families Bromeliaceae, Onagraceae, and Lamiaceae [22,25]; in the tribe Antirrhineae (Scrophulariaceae; [32]); and in the genus *Scrophularia* (Scrophulariaceae; [28]). Hexose-dominated nectars are prevalent in the Asteraceae, Solanaceae, Verbenaceae, Apiaceae, and Liliaceae families [22,25,27] and the Gentianales order [26]. In the subfamily Alooideae (Asphodelaceae), similar nectar traits were reported within its genera [33]. Furthermore, in the Proteaceae family, sucrose nectars are produced by some genera, whereas hexose nectars are produced by other genera [34]. A low but significant phylogenetic signal in nectar composition was also detected in a meta-analysis, covering more than 1000 species from 20% of all angiosperm families [17]. Nevertheless, in some plant lineages, plant phylogeny seems to be a weaker determinant of nectar composition than pollinator feeding preferences [35,36]. Moreover, some conclusions at the family level are controversial. For example, plants within the Fabaceae family have been reported to produce both sucrose [22] and hexose [25] nectars.

The abovementioned variability in the relative effects of pollinator-related selection and plant phylogeny on nectar properties indicates that the general patterns are rather complex. Consequently, we need comprehensive studies from various flora around the world. Unfortunately, we are still missing sufficient data from large biogeographic regions, such as the Afrotropics. This can be demonstrated by one of the most extensive compilations of nectar traits–pollinator group relationships to date, which extracted data from 53 studies [17]. Among these, there were only six studies from sub-Saharan Africa. Moreover, these six studies all focused on South African plants (however, see some data for Ethiopia in Schmidt-Lebuhn et al. [26]) and a single study targeted plants growing in tropical Africa. In this study, we aimed to contribute to addressing this gap by presenting the nectar properties of tropical plants growing in the rainforests of Mt. Cameroon (West-Central Africa). Our additional aim was to test for significant associations between pollinators and nectar properties among Mount Cameroon flora. Specifically, we asked the following three research questions: (1) Is nectar trait evolution associated with different pollinator groups? (2) Is the nectar of sunbird-pollinated plants growing in Tropical Africa rich in sucrose, as has been observed in other parts of the world? (3) What is the phylogenetical signal in nectar properties inside the studied plant lineages?

## 2. Results

Altogether, we analyzed the carbohydrate compositions of 803 nectar samples from the 66 target plant species on Mount Cameroon (Table 1). Sucrose was the most abundant carbohydrate in the nectars of the examined plants (sucrose: mean proportion ± SD = 54 ± 0.27%, 24 h mean production per flower = 0.36 ± 0.56 mg; glucose: mean proportion = 22 ± 0.14%, 24 h mean production per flower = 0.10 ± 0.17 mg; fructose: mean proportion = 24 ± 0.16%, 24 h mean production per flower = 0.18 ± 0.71 mg). Of the 66 studied species, more than half (37 species) had a sucrose/hexose ratio higher than 1 (mean = 3.01 ± 4.53).

**Figure 1:**
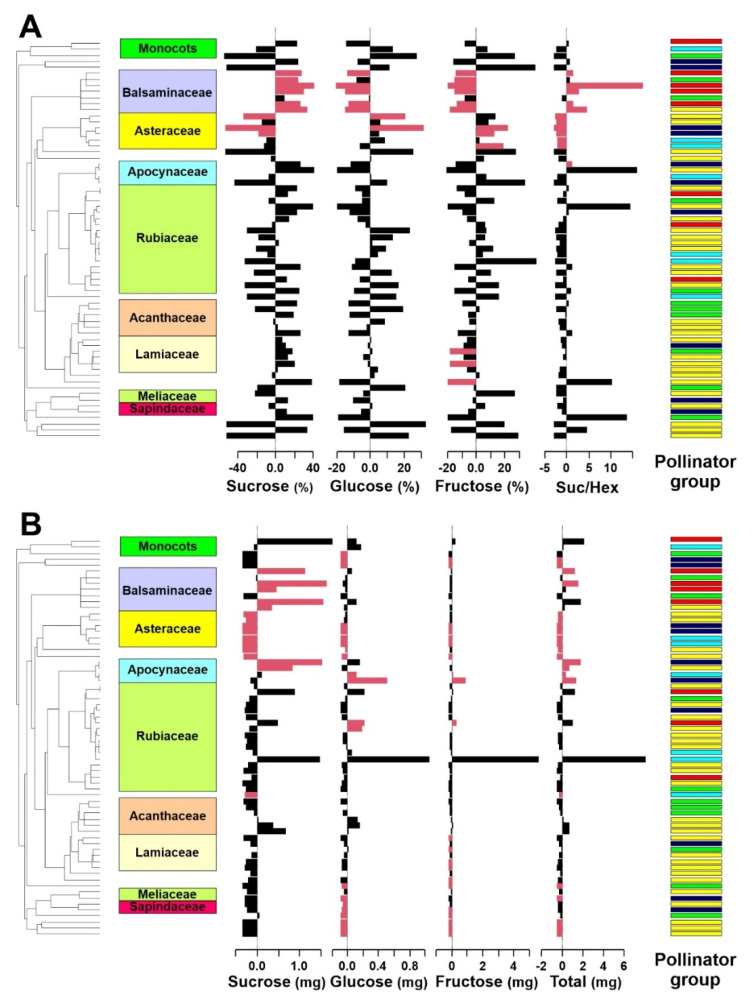
Phylogenetic signals in nectar traits along the phylogeny of plants on Mount Cameroon. (**A**) Proportion of individual sugars. (**B**) Amount of nectar sugar production per flower in 24 h. Each bar represents one plant species. The bars show centered values; therefore, the right- and left-facing bars represent values higher and lower than the mean across all plants, respectively. Red indicates species with significant local Moran’s I values (*p* < 0.05). Pollinator groups: yellow, bees; red, birds; light blue, butterflies; dark blue, moths; green, hoverflies. For a similar figure reporting individual species, see Supplementary Data Figure S1.

**Figure 2:**
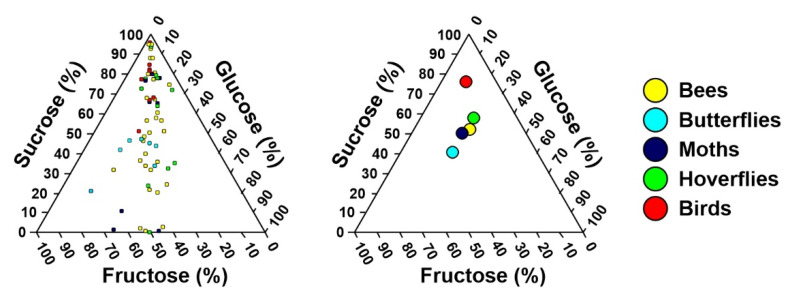
Nectar compositions of the studied plants visited by different groups of nectar-feeding pollinators on Mount Cameroon, with individual plants on the left and group means on the right.

A significant phylogenetic signal in the proportion of individual sugars was detected via most of the calculated indices (Table 2). In contrast, no significant phylogenetic signal was detected in the sucrose/hexose ratio. Using the local Moran’s I index, we detected a local phylogenetic signal mainly in two plant families, Balsaminaceae and Asteraceae (Figure 1). The mean proportions of sucrose, glucose, and fructose in Balsaminaceae were 82%, 10%, and 8%, respectively, whereas in Asteraceae, they were 31%, 33%, and 36%, respectively. The nectar composition differed between at least two plant groups with different pollinators (pMANOVA, *p* = 0.047; Figure 2). The group different from the others was the bird-pollinated plants since the Games–Howell post hoc tests detected differences between sunbirds and bees and sunbirds and butterflies. The nectar of the bird-pollinated plants had, on average, the highest proportion of sucrose and the lowest proportion of glucose and fructose (Figure 2). The sucrose/hexose ratio did not differ among the pollinator groups (pANOVA, *p* = 0.5162). Evolutionary modeling showed a similar pattern, with the best models for proportions of individual sugars being the OU-PG(2) models that assumed two optima: one for birds and one for insects (Table 3, Supplementary Table S3).

**Figure 3:**
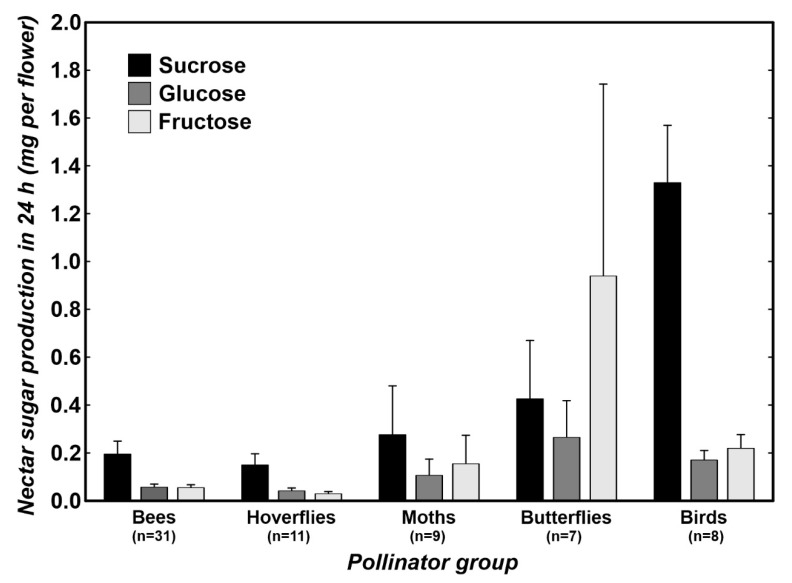
Production of individual sugars per flower in 24 h by plants visited by particular pollinator groups on Mount Cameroon. Mean ± SE. *n* = number of plant species per pollinator group.

We detected a significant phylogenetic signal in three of the four indices in the amount of sucrose (Table 2). In contrast, except for the glucose amounts, by considering Moran’s I, no significant phylogenetic signal was detected in the amounts of glucose or fructose. We did not find any phylogenetic signal in the total sugar amounts (Table 2). Local phylogenetic signals were detected in various families (Figure 1B). Regarding nectar composition, by considering the sugar amounts of individual carbohydrates, we found significant differences at least between the two groups of pollinators, but the differences were marginally statistically insignificant when considering the phylogeny (pMANOVA, *p* = 0.062; Figure 3). The most specific nectar composition was detected for the plants pollinated by birds with a high mean amount of sucrose (Figure 3). Using the Games–Howell post hoc tests, we detected significant differences in the amounts of all sugar types between birds and bees and birds and hoverflies. Plants pollinated by different pollinator groups differed in total sugar production (pANOVA, *p* = 0.020). The Games–Howell post hoc tests showed that the plants pollinated by birds produced significantly higher total sugar amounts than the plants pollinated by bees, hoverflies, or moths. Similar patterns were visible using the evolutionary models. For the sucrose amount and total carbohydrate amount evolution OU-PG(2) was the best fitting model assuming different evolution under the bird and insect selection pressures. In contrast, the glucose and fructose amounts were best fitted using an OU1 model assuming that these nectar traits evolved independently of the pollinator groups (Table 3, Supplementary Table S3).

## 3. Discussion

### 3.1. Differences in Nectar Properties in Relation to the Main Pollinator

The relatively higher proportion of sucrose than hexoses in the analyzed nectars corresponded with the results of the recent large compilation studies, demonstrating that nectars are generally more often dominated by sucrose [15,17]. We found that whereas the studied insect-pollinated plants had similar compositions of nectar, they differed from ornithophilous plants. The nectars of the plants pollinated by sunbirds were characterized by a high proportion of sucrose. Nectars with such a character were found not only in a few studies of plants pollinated by sunbirds [29,30] but also in several studies of hummingbird-pollinated plants [17,36,37,38]. Using the largest dataset from tropical Africa to date, our study confirmed that the plants pollinated by specialized birds also produce sucrose nectars in this region hosting the highest sunbird diversity. Unfortunately, we did not record any plants pollinated by generalized passerine birds, which should produce hexose nectars [39]. As a consequence, we could only partly confirm the hypothesis that there was no dichotomy in the nectar composition between plants pollinated by hummingbirds and passerine birds but that there was a difference in the nectar of plants pollinated by specialized and generalized birds [29].

We should be careful when interpreting the high sucrose content in the ornithophilous plants as an adaptation of the plant to ensure bird pollination. The possibility that animals adapt their foraging behavior or dietary requirements can also play a role [15]. This point of view is nevertheless not clearly supported by experimental choice studies. Hummingbirds and sunbirds preferred sucrose in some experiments [40,41] but not in others or only under specific sugar concentrations [42,43,44]. In addition, assimilation by specialized nectarivorous birds seemed to be similar for all three sugar types [40,45]. It is even more challenging to conclude why ornithophilous plants produce sucrose-dominant nectar when considering that field observation studies often report sunbirds feeding on a wide spectrum of plants other than ornithophilous plants [46,47,48].

Similar controversy can also be found in the nectars of entomophilous plants and insect preferences demonstrated by choice experiments. For example, some case studies have suggested that honeybees [49,50] and hawkmoths [51] preferred sucrose over hexoses, whilst butterflies [52] have been shown to avoid glucose in nectar. Such results were in agreement with the nectar compositions reported for melittophilous, sphingophilous, and psychophilous plants in some studies [23,26], although not always [25,27]. Consequently, the link among nectar composition, plant adaptations, pollinator adaptations, pollinator diet, and pollinator preferences is not clear and should be explored in future studies.

However, we must admit that our study has its limits. We were not able to test some other suggested patterns in nectar trait evolution mediated by different pollinator groups. For example, our data did not allow us to test the dichotomy between nectar traits of plants visited by generalized vs. specialized visitors [15] due to a lack of plants pollinated by generalized passerines in our data and the absence of morphological data on individual insect pollinators.

### 3.2. Phylogenetic Signal

Exploration of phylogenetic signals throughout the plant phylogeny revealed a significant local signal in Balsaminaceae and Asteraceae. The observed nectar traits were in accordance with other studies showing the prevalence of sucrose-dominant nectars in Balsaminaceae [18,30,53] and hexose-dominant nectars in Asteraceae [38,54]; however, see [18,22] for less consistent results. Sucrose-dominant nectar with a weaker local phylogenetic signal was also observed in Lamiaceae in our study, which is consistent with the findings of previous studies [22,37,55]. For the other families represented by more species in our dataset, we found, similar to other scholars, variable nectar composition in Rubiaceae [37,38] and Acanthaceae [23]. In contrast to other studies [18,37,38], we did not observe the prevalence of sucrose-dominant nectars in Apocynaceae.

## 4. Materials and Methods

### 4.1. Study Site

Our study was performed in the tropical forests of Mount Cameroon, the highest mountain in West-Central Africa and a regional biodiversity hotspot [56,57]. Data were collected on its southwestern slope along a tourist trail leading from the Mann’s Spring camp (2200 m a.s.l.) to Bakingili village at the seashore during nine expeditions between 2016 and 2020. During these expeditions, flowers of currently flowering zoophilous plants from all vegetation layers, including canopies, were sampled for nectar and the flower visitors were recorded.

### 4.2. Nectar Collection and Analyses

We measured 24 h of nectar production of individual plant species, with one sample collected from one individual of each species. In this study, we included 66 plant species for which we were able to collect and analyze at least three samples of nectar (i.e., the species for which we sampled at least three individual plants; Table 1). One or more randomly selected flowers were bagged for 24 h in mesh to prevent visitors’ access. After this period, the mesh bag was removed, and all nectar produced was collected. The methods of nectar collection depended on nectar production. When the plant produced enough nectar, we collected it using a microcapillary tube or a Hamilton syringe. Subsequently, we measured the nectar concentration using a Pal-1 pocket refractometer (Atago Co., Tokyo, Japan), allowing for calculations of the total sugar amount per flower. After each measurement, a nectar subsample for analyses of sugar composition was absorbed onto a filter paper and dried using silica gel. In a laboratory, the filter papers were washed with distilled water in an Eppendorf tube, and the sucrose and hexoses (i.e., fructose and glucose) were determined using a high-performance anion exchange chromatography pulsed amperometric detector (HPAE-PAD) with a Dionex ISC-3000 system. The sugars were separated using a CarboPac PA1 analytical column (Dionex, Sunnyvale, CA, USA). When the nectar production was low, we washed the flowers with distilled water using a Hamilton syringe. These samples were then diluted with ethanol to achieve a ±50% ethanol concentration. In the field, the samples were boiled for 15 min to deactivate enzymes [58]. In the laboratory, these samples were dried and transferred into a constant volume of distilled water. To determine the total sugar amounts, as well as particular sugar proportions, the samples were analyzed using HPAE-PAD as well. Note that we were not able to determine the sugar concentration for plants producing small nectar amounts, which is why we did not include the nectar sugar concentration in any analyses in this study. Nevertheless, data on sugar concentrations of some plant species are available in Table 1.

### 4.3. Recording Visitors

Floral visitors of the target plant species were observed in five replicates (i.e., five plant individuals) for 24 h each (i.e., 120 h per species). For the observation, we used security cameras with IR night vision (Vivotek IB8367T). In some cases, the recording period differed slightly because of technical errors or flower senescence. More details of the camera settings are described in Klomberg et al. [46]. The video recordings were checked either using Motion Meerkat 2.0.5 motion-detection software [59] when conditions allowed for it or manually through sped-up playback. For the purpose of this study, only potential pollinators touching plant reproductive organs (anthers and/or stigmas) and drinking nectar were considered. These visitors were classified into pollinator groups that are commonly recognized in the pollination syndrome concept [21,60,61]. Each plant was affiliated with the particular pollinator group with the highest visitation frequency, compared to other visiting pollinator groups (Supplementary Table S1). The visitation frequency (VF) of a particular pollinator group on the plant species was calculated using $VF=v_{tot}/\sum_{i}^{n}t_{i}$, where *v_tot_* was the total number of observed visits by all members of this group, *n* was the number of observed flowers and *t_i_* was the observation time of *i*th flower. We excluded pollinator groups and affiliated plants if the group was the primary visitor of fewer than five observed plant species. In this way, we ruled out three plants pollinated by unspecialized flies, one by bats, and one by sphingids. Consequently, our dataset comprised plants with visitors belonging to five pollinator groups: bees, butterflies, moths (excluding Sphingidae, for which a distinct pollination syndrome was defined; [61]), hoverflies, and birds.

### 4.4. Phylogenetic and Statistical Analyses

A phylogenetic tree was created by pruning the dated ALLMB Spermatophyta tree [62]. Species missing from the ALLMB tree were replaced prior to the analyses by a species that was as closely related as possible (Supplementary Table S2). Multichotomies were resolved randomly using the multi2di function in the ape package [63] in R 4.0.2 [64]. The phylogenetic signal for individual studied parameters was calculated in the phylosignal R package [65].

Of the multiple commonly used tree-level indices of phylogenetic signals [66], we decided to report four (Abouheif’s Cmean, Moran’s I, Blomberg’s K, and Pagel’s λ) to make this study comparable with most of the others. For more details on the differences among these measures of phylogenetic signals, see Münkemüller et al. [66]. To detect the hotspots of local phylogenetic signals (i.e., hotspots of local trait autocorrelation), we calculated the local Moran’s I for each tip of the tree. The significance of the tree-level phylogenetic signal and local Moran’s I were tested using randomization [65]. To explore the relationship between pollinator groups and nectar properties, we used two approaches, namely, the phylogenetic ANOVA/MANOVA approach and evolutionary modeling. Using the first approach, the differences between groups of plants with particular pollinators in nectar proportion and amount compositions were tested using phylogenetic MANOVA. The between-group differences in univariate traits (sucrose/hexose ratio, sugar production) were tested using phylogenetic ANOVA. The phylogenetic MANOVA and ANOVA analyses were based on simulations [67]. These analyses were performed in R using the geiger package [68].

We fitted the evolutionary models based on a “random walk,” where the trait values are changing randomly, i.e., Brownian motion (BM models; [69]) and Ornstein–Uhlenbeck (OU) models, which include, except the stochastic part (σ2), the parameter describing the strength of selection (α) and parameters determining one or more optima (θ) [70,71]. Specifically, we compared five models. (1) BM model, which assumes that the nectar traits evolved independently of the pollinator groups, that the directions of the changes were random, and that the rates of the changes were constant in all lineages. (2) BMS model, which was similar to the BM model, but the rates of the changes (σ2) differed between the pollinator groups. (3) OU1 model assuming that the nectar traits evolved independently of the pollinator groups, but the changes were directional toward a single optimum. (4) A OU-PG(5) model that allowed the evolution of nectar traits to head toward the optima, which differed for each of our five target pollinator groups. The strength of selection and the stochastic part were the same for all selection regimes. (5) OU-PG(2), i.e., the same model as OU-PG(5) but with only two optima: one for birds and one for insect pollinators. To map the ancestral stage along the phylogeny, we used the make.simmap function in the phylotools R package [72]. The models were fitted using the OUwie function in the OUwie R package [73].

## 5. Conclusions

Our study demonstrated that the effects of the main pollinator and phylogenetic signals were specific for individual plant groups. Not all groups of plants with the same main pollinator had typical nectar characteristics, and not all plant phylogenetic lineages showed phylogenetic signals in nectar traits. In our study, we found the main differences in nectar trait evolution between insect- and bird-pollinated plants. Our study on nectar trait evolution, which is the first from tropical Africa, supports some of the patterns found in other regions around the world.

## Figures and Tables

**Table 1:** Number of analyzed samples, nectar concentration, proportions of individual sugars in total sugar mass, and total sugar amount per flower. Con.—concentration. Means or mean ± SD are presented. Nectar samples were often collected from several flowers of one individual plant and as a consequence, the SD represents the variability among samples (plant individuals).

Species	No. of Samples	Con. (% *w*/*w*)	Sucrose (%)	Glucose (%)	Fructose (%)	Sugar Amount (µg/Flower)
*Acanthonema strigosum* Hook.f.	5	8.97	93.0 ± 5.7	3.7 ± 2.6	3.3 ± 3.2	150.5 ± 76.5
*Acanthopale decempedalis* C.B.Clarke	15	6.93	73.1 ± 9.6	9.7 ± 4	17.2 ± 5.7	386.1 ± 220.6
*Aframomum* sp. “purple”	38	17.71	34.0 ± 25.3	35.0 ± 11.6	31.1 ± 15.6	763 ± 863
*Argocoffeopsis afzelii* (Hiern) Robbr.	4		35.5 ± 8.9	35.1 ± 1.1	29.4 ± 9.7	120.8 ± 90.7
*Baccharoides calvoana* (Hook.f.) “Isawumi, El-Ghazaly & B.Nord.”	14	44.44	39.9 ± 6.4	27.9 ± 3.7	32.2 ± 3.7	189.9 ± 110.6
*Bertiera racemosa* (G.Don) K.Schum.	3	18.43	24.1 ± 4.7	45.1 ± 4.7	30.7 ± 7.8	628.3 ± 476.5
*Brillantaisia owariensis* P.Beauv.	24	30.2	56.9 ± 10.3	19.7 ± 8	23.4 ± 5.7	1295.3 ± 1024.4
*Calochone acuminata* Keay	4	20.75	21 ± 6.8	13.2 ± 0.2	65.7 ± 6.9	8742.7 ± 3191.5
*Chlorophytum comosum* (Thunb.) Jacques	6		0.2 ± 0.2	49.5 ± 7.2	50.3 ± 7.2	27.2 ± 19.7
*Clematis simensis* Fresen.	3		78.1 ± 14.8	14.5 ± 10.6	7.3 ± 4.2	9.4 ± 1.6
*Clerodendrum silvanum* Henriq.	17	11.69	65.3 ± 15.5	20.4 ± 8.3	14.2 ± 8.2	279.2 ± 210.2
*Costus dubius* (Afzel.) K.Schum.	7	18.6	77.2 ± 20	7.6 ± 9.2	15.3 ± 13.7	2755.6 ± 3435
*Crassocephalum montuosum* (S.Moore) Milne-Redh.	6		36.5 ± 26.6	27.5 ± 16.7	36 ± 15.1	13.5 ± 7.4
*Cuviera longiflora* Hiern	3		51 ± 14.3	19.5 ± 5.7	29.4 ± 8.6	1627.3 ± 616.9
*Deinbollia* sp. 1	15		66.1 ± 21.7	16.4 ± 12.1	17.5 ± 10.3	164.5 ± 119.1
*Dicranolepis vestita* Engl.	6	20.25	94.3 ± 2.9	2.8 ± 1.5	2.9 ± 1.5	422.2 ± 283.8
*Dioscoreophyllum cumminsii* (Stapf) Diels	4		1.7 ± 3.3	33.3 ± 17.7	65 ± 14.7	4.3 ± 2.4
*Dischistocalyx strobilinus* C.B.Clarke	31	17.03	50.1 ± 19.7	30 ± 10.5	19.9 ± 13	617.4 ± 595.6
*Discoclaoxylon hexandrum* (Müll.Arg.) Pax & K.Hoffm.	8		88.2 ± 8	6.5 ± 4.8	5.4 ± 3.4	7.4 ± 5.3
*Discopodium penninervium* Hochst.	5		23.5 ± 8.9	37.3 ± 4.2	39.3 ± 5.1	238.7 ± 162.2
*Distephanus biafrae* (Oliv. & Hiern) H.Rob.	5		20.7 ± 12.3	42.7 ± 7.3	36.6 ± 6.7	154.7 ± 94.2
*Gomphia flava* Schumach. & Thonn.	5		2.6 ± 2.1	54.4 ± 2.6	43 ± 3.3	7.7 ± 4.9
*Heckeldora staudtii* (Harms) Staner	5		67.1 ± 11.8	11.9 ± 3.1	21 ± 8.9	74.7 ± 19.2
*Heinsia crinita* (Afzel.) G.Taylor	3	23.5	46.8 ± 28.6	17.3 ± 14.8	35.9 ± 28.9	352.7 ± 143.3
*Hypoestes triflora* (Forssk.) Roem. & Schult.	20	17.88	32.6 ± 11.5	41.4 ± 9.1	26 ± 5.8	229.6 ± 164
*Ilex mitis* (L.) Radlk.	7	37.9	48.8 ± 10.7	22.6 ± 5	28.6 ± 6.3	78.2 ± 92.9
*Impatiens burtonii* Hook.f.	18	30.82	78.3 ± 19.4	14.1 ± 12.7	7.6 ± 7.2	423.3 ± 358.1
*Impatiens frithii* Cheek	11	16.12	85 ± 10.4	7.2 ± 5	7.8 ± 5.6	957.3 ± 645
*Impatiens hians* Hook.f.	20	19.75	95.3 ± 5.6	1.7 ± 1.9	2.9 ± 4.1	2107.7 ± 1758.9
*Impatiens macroptera* Hook.f.	25	26.01	88.2 ± 21	6.9 ± 13	4.9 ± 8.2	786.5 ± 691.9
*Impatiens mannii* Hook.f.	13		64 ± 35.5	21 ± 20.4	15.1 ± 15.1	59.1 ± 66.3
*Impatiens niamniamensis* Gilg	46	15.95	82 ± 16.8	8.8 ± 8.5	9.2 ± 8.9	1819.7 ± 1530.4
*Impatiens sakeriana* Hook. f.	7	19.7	81.4 ± 14.9	9 ± 7.3	9.6 ± 7.8	2345.4 ± 1583.2
*Isodon ramosissimus* (Hook.f.) Codd	14	15.62	56.8 ± 10.6	26.3 ± 6.9	16.9 ± 4	100.5 ± 30.6
*Isoglossa glandulifera* Lindau	10	6.5	77.1 ± 9.1	9.2 ± 5	13.7 ± 6.3	43.4 ± 25.3
*Ixora foliosa* Hiern	9	13.88	94.6 ± 1.9	2.4 ± 1	3 ± 1.1	98 ± 34.2
*Ixora guineensis* Benth.	18	12.09	76.8 ± 17.7	9.7 ± 8.4	13.5 ± 11.1	59 ± 58.2
*Laccodiscus ferrugineus* (Baker) Radlk.	17		46.8 ± 36.4	23.5 ± 18.9	29.7 ± 21.3	137 ± 123.2
*Melanthera scandens* (Schumach. & Thonn.) Roberty	13		44 ± 23.2	30.8 ± 16.9	25.2 ± 11	13.1 ± 14.1
*Mikania cordata* (Burm.f.) B.L.Rob.	15		41.8 ± 20.2	15.9 ± 8.2	42.4 ± 14	5.6 ± 4.1
*Nuxia congesta* R.Br. ex Fresen.	5	23.06	50.4 ± 4.5	24.4 ± 3	25.2 ± 1.9	386.3 ± 117.7
*Oncoba dentata* Oliv.	3		2.4 ± 2.1	44.5 ± 8.9	53 ± 9.1	7.6 ± 3.4
*Pavetta hookeriana* Hiern	9	31.58	33.9 ± 13	31 ± 6.3	35.1 ± 7.3	244.4 ± 218.1
*Pavetta neurocarpa* Benth.	15	23.68	58 ± 12.4	23.5 ± 7.5	18.4 ± 5.9	185.1 ± 103.9
*Pavetta rigida* Hiern	19	12.36	45.2 ± 11.3	26.8 ± 5.6	28 ± 10	562.8 ± 549.5
*Plectranthus decurrens* (Gürke) J.K.Morton	20	19.21	74.6 ± 8.7	20.8 ± 6	4.7 ± 3.4	121 ± 71.3
*Plectranthus glandulosus* Hook.f.	15	36.05	67.4 ± 12.3	18.2 ± 5.3	14.4 ± 7.3	320.9 ± 160.7
*Plectranthus kamerunensis* Gürke	18	29.24	72.3 ± 8.8	23.3 ± 7.2	4.4 ± 2.4	478.5 ± 353.3
*Psychotria bifaria* Hiern	10		21.9 ± 32.2	38.8 ± 15.2	39.3 ± 17.6	58.3 ± 57.5
*Psychotria leptophylla* Hiern	11		31.6 ± 35.3	34.6 ± 17.7	33.8 ± 17.7	101.6 ± 40.5
*Psychotria peduncularis* (Salisb.) Steyerm.	16	14.13	66.7 ± 17	16 ± 6.8	17.3 ± 10.7	332 ± 324.5
*Psychotria thonneri* (De Wild. & T. Durand) O. Lachenaud	7	9.9	80.8 ± 16.8	11.4 ± 11.4	7.8 ± 5.7	182.4 ± 129.7
*Psydrax dunlapii* (Hutch. & Dalziel) Bridson	3	11.5	68.4 ± 2.9	14.5 ± 2	17.2 ± 1	102.1 ± 32.6
*Sabicea calycina* Benth.	15	24.35	77.2 ± 22.8	13 ± 8.7	9.9 ± 17.1	346.8 ± 234.7
*Sabicea pilosa* Hiern	16	18.34	68 ± 17.8	17.1 ± 9.3	14.9 ± 9.5	1817.5 ± 1771.8
*Schefflera abyssinica* (Hochst. ex A.Rich.) Harms	5	11.12	1 ± 0.9	47.4 ± 0.9	51.6 ± 1.2	127.9 ± 145.8
*Solanecio mannii* (Hook.f.) C.Jeffrey	5		0.8 ± 1	53.3 ± 3.5	46 ± 4.2	8.6 ± 4.6
*Spermacoce princeae* (K.Schum.) Verdc.	5	11	79.8 ± 14.6	12.5 ± 7.8	7.7 ± 7.7	98.6 ± 38.4
*Stachys aculeolata* Hook.f.	13		61 ± 16.5	22.6 ± 9.2	16.4 ± 7.7	42.4 ± 28.9
*Stellaria mannii* Hook.f.	14		35.3 ± 15.2	42.7 ± 9.1	22 ± 7.8	37 ± 27
*Tabernaemontana brachyantha* Stapf	6	11.6	10.6 ± 18.1	32 ± 7.4	57.4 ± 12.9	1894.5 ± 1344.6
*Tabernaemontana ventricosa* Hochst. ex A.DC.	18	16.84	46.9 ± 29.5	22.4 ± 12.2	30.7 ± 20.6	980.6 ± 873.7
*Thunbergia fasciculata* Lindau	7	13.68	80.4 ± 9.4	8.9 ± 3.8	10.7 ± 7.5	1271.1 ± 2178.7
*Trichilia rubescens* Oliv.	22	22.02	32 ± 23	17.9 ± 13.9	50.1 ± 19.8	312.9 ± 305.8
*Voacanga africana* Stapf ex Scott-Elliot	15	14.7	95 ± 4.1	2.3 ± 1.9	2.7 ± 2.4	1239.4 ± 575.8
*Voacanga bracteata* Stapf	12	16.69	80.4 ± 21.5	10.6 ± 13.6	8.9 ± 8.3	2354 ± 2817.9

**Table 2:** Phylogenetic signal measured by four indices for individual nectar sugar parameters.

	Abouheif’s C_mean_	Moran’s I	Blomberg’s K	Pagel’s λ
**Sucrose (%)**	0.132 *	0.057 *	0.250 **	0.596 **
**Glucose (%)**	0.082	0.034 ^†^	0.256 **	0.490 ^†^
**Fructose (%)**	0.133 *	0.061 *	0.195 *	0.442 *
**Sucrose *amount***	0.185 *	0.056 *	0.080	0.329 *
**Glucose *amount***	0.091 ^†^	0.020 *	0.055	0.209
**Fructose *amount***	−0.001	−0.015	0.071	0.000
**Sucrose/hexose ratio**	0.038	0.009	0.097	0.000
**Sugar amount**	0.046	−0.004	0.069	0.000

**^†^** 0.1 > *p* > 0.05; * 0.05 > *p* > 0.01; ** *p* < 0.01.

**Table 3:** AICc values for evolutionary brown motion (BM) and Oornstein–Uhlenbeck (OU) models explaining individual nectar traits. The lowest AICc values indicating the best-fitting model are marked in bold. *pro.*—proportion.

	Sucrose Pro.	Glucose Pro.	Fructose Pro.	Suc/Hex Ratio	Total Amount	Sucrose Amount	Glucose Amount	Fructose Amount
**BM1**	57.51	8.06	35.84	462.94	311.64	200.03	69.37	238.24
**BMS**	47.04	−21.31	12.35	442.54	301.15	180.20	60.74	211.51
**OU1**	15.79	−70.48	−40.70	389.88	218.48	116.47	**−40.60**	**145.58**
**OU-PG_(5)_**	16.76	−64.36	−46.73	398.28	215.98	117.30	−39.36	152.53
**OU-PG_(2)_**	**10.04**	**−75.11**	**−55.33**	**388.16**	**214.37**	**77.60**	−39.23	147.82

## Data Availability

Data are presented in Table 1 and Table S1.

## Supplementary Materials

The following are available online at https://www.mdpi.com/article/10.3390/plants10061161/s1, Table S1: The main pollinators of individual plant species and their visitation frequencies. Table S2: The replacement of some species missing in the ALLMB Spermatophyta tree (Smith and Brown 2018) by relatives. Table S3: Parameters of models for individual nectar traits with the best fits. Figure S1: Similar to Figure 1 in the main text but showing the phylogenetic positions of individual species.
