# Supplementary material for: Differences in Nectar Traits between Ornithophilous and Entomophilous Plants on Mount Cameroon
Source: Plants (Basel). 2021 Jun 8;10(6):1161. doi: 10.3390/plants10061161 (PMC8226534; doi:10.3390/plants10061161)
Supplement: Supplementary file 1 [file plants-10-01161-s001.zip › plants-1243118-supplementary.pdf]

Table S1. The main pollinators of individual plant species and their visitation frequencies.

Plant species	Plant family	Pollinator group	Visitation frequency (flower^{-1},hr^{-1})	The proportion of visits by the main pollinator group (%)
Acanthonema strigosum	Gesneriaceae	Bee	0.0676	70.9
Acanthopale decempedalis	Acanthaceae	Hoverfly	0.0306	100
Aframomum sp purple	Zingiberaceae	Butterfly	0.1778	57.7
Argocoffeopsis afzelii	Rubiaceae	Bee	0.0149	46.2
Baccharoides calvoana	Compositae	Bee	0.3773	81.1
Bertiera racemosa	Rubiaceae	Bee	1.0633	99.4
Brillantaisia owariensis	Acanthaceae	Bee	0.1019	95.7
Calochone acuminata	Rubiaceae	Butterfly	0.1211	100
Chlorophytum comosum	Asparagaceae	Hoverfly	0.1617	100
Clematis simensis	Ranunculaceae	Moth	0.0647	59.5
Clerodendrum silvanum	Lamiaceae	Moth	0.0581	64.8
Costus dubius	Costaceae	Passeriformes	0.0915	75
Crassocephalum montuosum	Compositae	Bee	0.3074	44.5
Cuviera longiflora	Rubiaceae	Passeriformes	0.0293	66.5
Deinbollia sp 1	Sapindaceae	Moth	0.0988	55.6
Dicranolepis vestita	Thymelaeaceae	Hoverfly	0.0207	42.9
Dioscoreophyllum cumminsii	Menispermaceae	Moth	0.0528	100
Dischistocalyx strobilinus	Acanthaceae	Bee	0.5958	56.4
Discoclaoxylon hexandrum	Euphorbiaceae	Bee	0.0193	47.9
Discopodium penninervium	Solanaceae	Hoverfly	0.2074	80
Distephanus biafrae	Compositae	Bee	0.0913	38
Gomphia flava	Ochnaceae	Bee	0.0302	85.1
Heckeldora staudtii	Meliaceae	Moth	0.0543	100
Heinsia crinita	Rubiaceae	Butterfly	0.0447	66.7
Hypoestes triflora	Acanthaceae	Hoverfly	0.0557	37.2
Ilex mitis	Aquifoliaceae	Bee	0.1940	82.9
Impatiens burtonii	Balsaminaceae	Hoverfly	0.3521	79.6
Impatiens frithii	Balsaminaceae	Passeriformes	0.0062	100
Impatiens hians	Balsaminaceae	Passeriformes	0.0304	100
Impatiens macroptera	Balsaminaceae	Bee	0.6715	66.6
Impatiens mannii	Balsaminaceae	Hoverfly	0.6804	86.7
Impatiens niamniamensis	Balsaminaceae	Passeriformes	0.0384	53.5
Impatiens sakeriana	Balsaminaceae	Passeriformes	0.0702	100
Isodon ramosissimus	Lamiaceae	Bee	0.1475	83.3
Isoglossa glandulifera	Acanthaceae	Hoverfly	0.1274	97.9
Ixora foliosa	Rubiaceae	Bee	0.0065	43.1
Ixora guineensis	Rubiaceae	Moth	0.2621	71.6
Laccodiscus ferrugineus	Sapindaceae	Bee	0.0919	88.6
Melanthera scandens	Compositae	Butterfly	0.1374	55.2
Mikania cordata	Compositae	Butterfly	0.0053	66.7
Nuxia congesta	Stilbaceae	Bee	0.1085	88.6
Oncoba dentata	Salicaceae	Bee	0.0845	100
Pavetta hookeriana	Rubiaceae	Bee	0.2768	82.6
Pavetta neurocarpa	Rubiaceae	Bee	0.0111	73.3
Pavetta rigida	Rubiaceae	Butterfly	0.0024	93.3
Plectranthus decurrens	Lamiaceae	Bee	0.5710	98.6
Plectranthus glandulosus	Lamiaceae	Bee	0.0910	65.7

Plectranthus kamerunensis	Lamiaceae	Hoverfly	0.1521	53.1
Psychotria bifaria	Rubiaceae	Bee	0.2458	84.7
Psychotria leptophylla	Rubiaceae	Bee	0.1673	44
Psychotria peduncularis	Rubiaceae	Passeriformes	0.2263	73.9
Psychotria thonneri	Rubiaceae	Bee	0.4177	67.6
Psydrax dunlapii	Rubiaceae	Bee	0.3555	69.3
Sabicea calycina	Rubiaceae	Bee	0.3729	96.7
Sabicea pilosa	Rubiaceae	Passeriformes	0.1553	90.9
Schefflera abyssinica	Araliaceae	Bee	0.8157	95.7
Solanecio mannii	Compositae	Moth	0.0116	95
Spermacoce princeae	Rubiaceae	Hoverfly	0.0805	70.1
Stachys aculeolata	Lamiaceae	Bee	0.2318	71.2
Stellaria mannii	Caryophyllaceae	Hoverfly	0.0255	85.7
Tabernaemontana brachyantha	Apocynaceae	Moth	0.0303	40.1
Tabernaemontana ventricosa	Apocynaceae	Butterfly	0.0543	23.4
Thunbergia fasciculata	Acanthaceae	Bee	0.4088	70
Trichilia rubescens	Meliaceae	Bee	0.0869	30.8
Voacanga africana	Apocynaceae	Bee	0.1457	46.1
Voacanga bracteata	Apocynaceae	Moth	0.0915	100

Table S2. The replacement of some species missing in ALLMB Spermatophyta tree (Smith and Brown 2018) by relatives. The checklist of plants included in this study "Species" and plants used in the phylogeny tree "Phylogenetical tree species". Substitutes are indicated by phylogenetic tree species names in bold.

Species	Phylogenetical tree species	family
Acanthonema strigosum	***Saintpaulia inconspicua***	*Gesneriaceae*
Acanthopale decempedalis	*Acanthopale decempedalis*	*Acanthaceae*
Aframomum sp purple	***Aframomum luteoalbum***	*Zingiberaceae*
Argocoffeopsis afzelii	***Calycosiphonia spathicalyx***	*Rubiaceae*
Baccharoides calvoana	***Baccharoides lasiopus***	*Asteraceae*
Bertiera racemosa	*Bertiera racemosa*	*Rubiaceae*
Brillantaisia owariensis	***Brillantaisia vogeliana***	*Acanthaceae*
Calochone acuminata	*Calochone acuminata*	*Rubiaceae*
Chlorophytum comosum	*Chlorophytum comosum*	*Asparagaceae*
Clematis simensis	*Clematis simensis*	*Ranunculaceae*
Clerodendrum silvanum	*Clerodendrum silvanum*	*Lamiaceae*
Costus dubius	*Costus dubius*	*Costaceae*
Crassocephalum montuosum	*Crassocephalum montuosum*	*Asteraceae*
Cuviera longiflora	*Cuviera longiflora*	*Rubiaceae*
Deinbollia sp 1	***Deinbollia macrocarpa***	*Sapindaceae*
Dicranolepis vestita	*Dicranolepis vestita*	*Thymelaeaceae*
Dioscoreophyllum cumminsii	*Dioscoreophyllum cumminsii*	*Menispermaceae*
Dischistocalyx strobilinus	*Dischistocalyx strobilinus*	*Acanthaceae*
Discoclaoxylon hexandrum	*Discoclaoxylon hexandrum*	*Euphorbiaceae*
Discopodium penninervium	*Discopodium penninervium*	*Solanaceae*
Distephanus biafrae	***Distephanus barus***	*Asteraceae*
Gomphia flava	***Gomphia densiflora***	*Ochnaceae*
Heckeldora staudtii	*Heckeldora staudtii*	*Meliaceae*
Heinsia crinita	*Heinsia crinita*	*Rubiaceae*
Hypoestes triflora	*Hypoestes triflora*	*Acanthaceae*
Ilex mitis	*Ilex mitis*	*Aquifoliaceae*

Impatiens burtonii subsp. burtonii	*Impatiens burtonii subsp. burtonii*	*Balsaminaceae*
Impatiens frithii	*Impatiens frithii*	*Balsaminaceae*
Impatiens hians	*Impatiens hians*	*Balsaminaceae*
Impatiens macroptera	*Impatiens macroptera*	*Balsaminaceae*
Impatiens mannii	*Impatiens mannii*	*Balsaminaceae*
Impatiens niamniamensis	*Impatiens niamniamensis*	*Balsaminaceae*
Impatiens sakeriana	*Impatiens sakeriana*	*Balsaminaceae*
Isodon ramosissimus	*Isodon ramosissimus*	*Lamiaceae*
Isoglossa glandulifera	*Isoglossa glandulifera*	*Acanthaceae*
Ixora foliosa	*Ixora foliosa*	*Rubiaceae*
Ixora guineensis	*Ixora guineensis*	*Rubiaceae*
Laccodiscus ferrugineus	*Laccodiscus ferrugineus*	*Sapindaceae*
Melanthera scandens	***Melanthera remyi***	*Asteraceae*
Mikania cordata	*Mikania cordata*	*Asteraceae*
Nuxia congesta	*Nuxia congesta*	*Stilbaceae*
Oncoba dentata	***Oncoba spinosa***	*Salicaceae*
Pavetta hookeriana	*Pavetta hookeriana*	*Rubiaceae*
Pavetta neurocarpa	*Pavetta neurocarpa*	*Rubiaceae*
Pavetta rigida	*Pavetta rigida*	*Rubiaceae*
Plectranthus decurrens	*Plectranthus decurrens*	*Lamiaceae*
Plectranthus glandulosus	*Plectranthus glandulosus*	*Lamiaceae*
Plectranthus kamerunensis	*Plectranthus kamerunensis*	*Lamiaceae*
Psychotria bifaria	*Psychotria bifaria*	*Rubiaceae*
Psychotria leptophylla	*Psychotria leptophylla*	*Rubiaceae*
Psychotria peduncularis	*Psychotria peduncularis*	*Rubiaceae*
Psychotria thonneri	***Psychotria ledermannii***	*Rubiaceae*
Psydrax dunlapii	*Psydrax dunlapii*	*Rubiaceae*
Sabicea calycina	*Sabicea calycina*	*Rubiaceae*
Sabicea pilosa	*Sabicea pilosa*	*Rubiaceae*
Schefflera abyssinica	*Schefflera abyssinica*	*Araliaceae*
Solanecio mannii	*Solanecio mannii*	*Asteraceae*
Spermacoce princeae	*Spermacoce princeae*	*Rubiaceae*
Stachys aculeolata	*Stachys aculeolata*	*Lamiaceae*
Stellaria mannii	*Stellaria mannii*	*Caryophyllaceae*
Tabernaemontana brachyantha	*Tabernaemontana brachyantha*	*Apocynaceae*
Tabernaemontana ventricosa	*Tabernaemontana ventricosa*	*Apocynaceae*
Thunbergia fasciculata	*Thunbergia fasciculata*	*Acanthaceae*
Trichilia rubescens	*Trichilia rubescens*	*Meliaceae*
Voacanga africana	*Voacanga africana*	*Apocynaceae*
Voacanga bracteata	*Voacanga bracteata*	*Apocynaceae*

Table S3. Parameters of models for individual nectar traits with the best fits (see Table 3). σ2-random drift; α – strength of selection; θBIRDS – adaptive peak for birds; θINSECTS – adaptive peak for insects.

	**Sucrose pro.**	**Glucose pro.**	**Fructose pro.**	**Suc/Hex ratio**	**Total amount**	**Sucrose amount**	**Glucose amount**	**Fructose amount**
**best-fitting model**	**OU-PG(2)**	**OU-PG(2)**	**OU-PG(2)**	**OU-PG(2)**	**OU-PG(2)**	**OU-PG(2)**	**OU1**	**OU1**
σ^2	0.014	0.086	0.129	10.333	6.922	0.140	0.068	0.243
α	0.106	2.614	2.614	0.267	2.613	0.406	1.167	0.238
θ_{BIRDS}	0.857	0.100	0.127	6.500	1.807	1.609	---	---
$\theta_{INSECTS}$	0.500	0.237	0.250	2.604	0.499	0.213	---	---

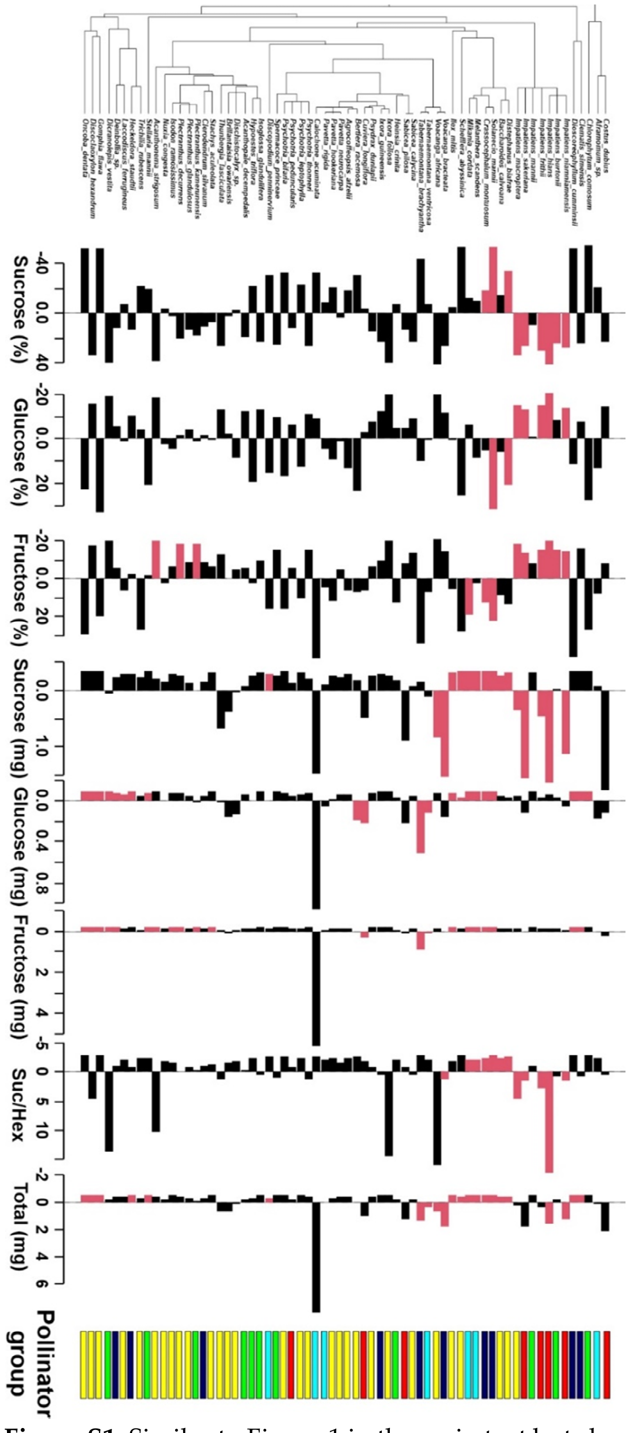

Figure S1. Similar to Figure 1 in the main text but showing the phylogenetic positions of individual species.
